# Subungual onycholemmal cysts presenting as longitudinal leukonychya: a case report and review of literature^؆^

**DOI:** 10.1016/j.abd.2026.501403

**Published:** 2026-06-23

**Authors:** María Paula Muñoz Mc Causland, Annie Mariana Meléndez Hernández, Juan Carlos López Hiromi, Isabela Dorado Caycedo

**Affiliations:** a Department of Dermatology, Faculty of Medicine, Fundación Universitaria Sanitas, Bogotá, Colombia; b Department of Dermatology, Faculty of Medicine, Fundación Universitaria Sanitas, Bogotá, Colombia; c Research Group IMPAC, Department of Pathology, Faculty of Medicine, Fundación Universitaria Sanitas, Bogotá, DC, Colombia; d Department of Dermatology, Centros Médicos Colsanitas, Bogotá, Colombia

Dear Editor,

Onychocytic cysts are uncommon, typically asymptomatic nail bed lesions, often detected incidentally in biopsies or excisions.^1^, ^2^ They can present with pachyonychia, onycholysis, hyperkeratosis, or nail dystrophy, making them difficult to distinguish from other neoplasms.^1^

We present a rare case of onycholemmal cysts presenting as longitudinal leukonychia and review its differential diagnoses.

A 36-year-old woman from Bogotá, Colombia, presented with a 10-year history of a right thumb nail lesion, occasionally associated with mild pain. She had no history of trauma, prior treatments, or relevant medical conditions. Examination revealed longitudinal leukonychia reaching the lunula, V-shaped onycholysis, and a distal fissure (Fig. 1). Dermoscopy of the free edge showed subtle subungual hyperkeratosis (Fig. 2), initially suggesting onychopapilloma. Due to persistent symptoms, surgical intervention was performed. Following nail plate avulsion, the nail bed appeared normal, while intraoperative dermoscopy revealed faint yellowish globules (Fig. 2). A longitudinal biopsy uncovered firm subungual tissue. Histopathology demonstrated mild acanthosis of the nail bed epithelium and multiple small onycholemmal cysts in the dermis (Fig. 3).

**Fig. 1:**
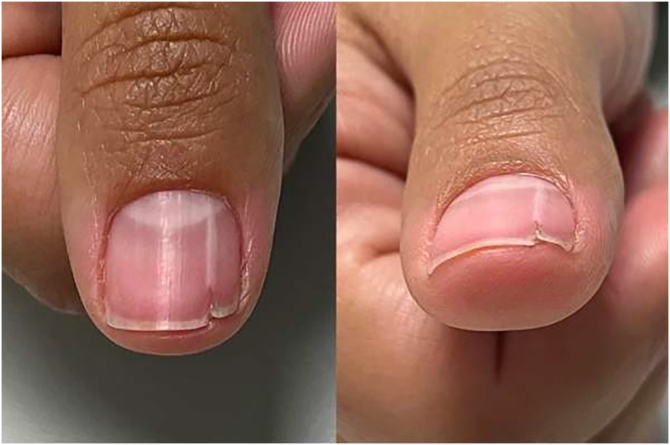
Longitudinal leukonychia extending to the lunula, V-shaped onycholysis, and a distal fissure.

**Fig. 2:**
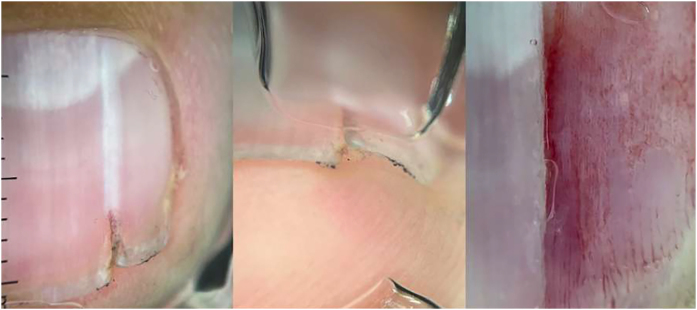
Dermoscopy of the free edge revealed discrete subungual hyperkeratosis. After nail plate avulsion, intraoperative dermoscopy showed very subtle yellowish globules.

**Fig. 3:**
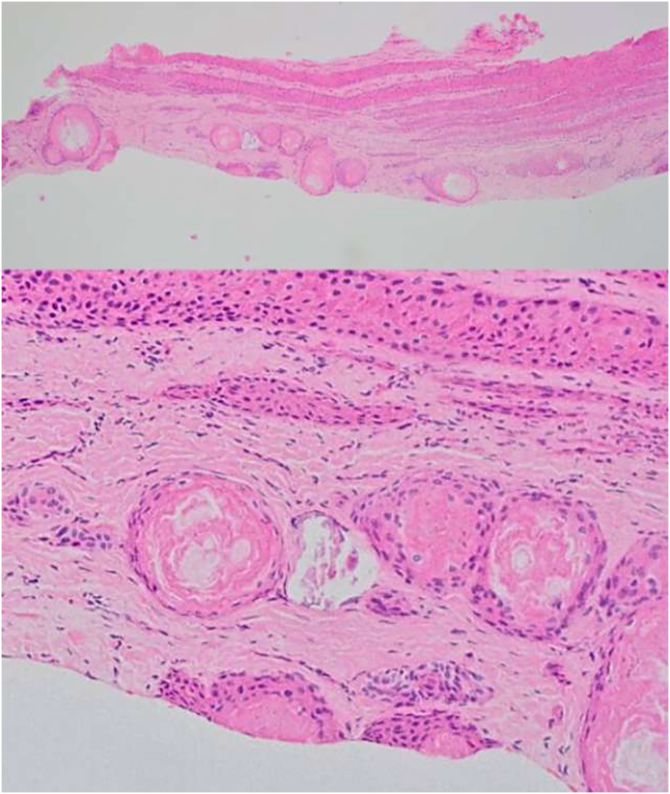
Nail bed epithelium with mild acanthosis, multiple small onycholemmal cysts in the dermis.

Longitudinal leukonychia can result from benign, malignant, or inflammatory nail disorders (Table 1). We report the first case of onycholemmal cysts presenting as longitudinal leukonychia.^1^, ^2^ Clinically, these cysts display variable features, including nail plate ridging, subungual hyperkeratosis, digital clubbing, onycholysis, or pincer nail deformity.^1^, ^2^ Their pathogenesis remains unclear: Samman (1959) suggested they result from epidermal implantation after trauma;^3^ in patients with clubbing, a dermal fibroblast proliferation has also been suggested.^2^ They likely originate from the squamous epithelium of the distal nail bed at the isthmus transition zone. Histologically, onycholemmal cysts are lined by keratinized stratified squamous epithelium lacking a granular layer, with laminated eosinophilic keratin and an eosinophilic cuticle resembling the outer root sheath of hair follicles.^1^, ^2^, ^3^

**Table 1:** Differential diagnoses of longitudinal leukonychia in one or multiple digits: clinical and histopathological characteristics.^1^, ^2^, ^3^, ^4^, ^5^, ^6^, ^7^, ^8^, ^9^, ^10^.

Diagnosis	Clinical Characteristics	Histopathology
	*Single Digit*	
Onychopapilloma	Leukonychia or erythronychia longitudinal, distal onycholysis, V-shaped fissures	Papillomatosis, acanthosis of the distal nail bed with premature keratinization, matrical metaplasia
Onychomatricoma	Longitudinal melanonychia or leukonychia, pachyonychia	Cystic spaces, hyperplasia of the matrix, monomorphic onychocytes
Onychocytic Matricoma	Longitudinal melanonychia or leukonychia, pachyonychia	Deep endokeratinization with concentric nests of prekeratogenous and keratogenous cells
Subungual Seborrheic Keratosis	Progressive leukonychia or melanonychia, distortion of the nail plate	Irregular hyperplasia of basaloid cells forming eosinophilic whorls
Squamous Cell Carcinoma	Leukonychia or erythronychia, distal onycholysis, pain	Epidermal dysplasia, atypical keratinocytes
Chronic Trauma	Leukonychia, usually in the hallux	Hyperkeratosis, irregular acanthosis, spongiosis, parakeratosis
	*Multiple Digits*	
Darier Disease	Leukonychia or erythronychia, “candy cane” nails, V-shaped nicks	Suprabasal acantholysis, dyskeratosis, hyperkeratosis, parakeratosis, epithelial hyperplasia of the matrix (white bands), matrix thinning (red bands)
Onychomicosis	Pseudo-leukonychia characterized by white or yellowish irregular streaks	Hyperkeratosis, parakeratosis, intrakeratonic neutrophils, PAS positive for fungal elements

Management lacks standardized guidelines.^1^, ^2^ Biopsy is diagnostic and therapeutic, allowing partial or complete excision.^1^ Recognizing underlying causes is essential, as leukonychia may occur in diverse contexts. This observation prompted a review of the potential causes of longitudinal leukonychia and its differential diagnosis. The pattern ‒ localized versus multifocal ‒ guides the differential diagnosis (Table 1).

## Single-digit leukonychia

Localized tumors or trauma are the main considerations. Benign tumors such as onychopapilloma may present as longitudinal erythronychia or, less commonly, as leukonychia or melanonychia, often accompanied by distal onycholysis, V-shaped fissures, or splinter hemorrhages. Histopathology typically shows papillomatosis, acanthosis of the distal nail bed, premature keratinization, and matrix metaplasia.^4^, ^5^

Onychomatricoma and onychocytic matricoma ‒ benign nail matrix tumors ‒ can manifest with pachyonychia, longitudinal melanonychia, or, occasionally, leukonychia. The former demonstrates cystic spaces and matrix hyperplasia with monomorphic onychocytes forming nests, whereas the latter shows endokeratinization in the deeper portion of the neoplasm and concentrically arranged nests of prekeratogenous and keratogenous cells.^5^, ^6^

Nail unit seborrheic keratosis may produce longitudinal melanonychia or, less frequently, leukonychia with progressive band widening and nail plate distortion. Histologically, it exhibits irregular hyperplasia of the distal matrix and nail bed, with basaloid cells forming eosinophilic whorls.^7^, ^8^

Malignant tumors, particularly squamous cell carcinoma, may also present as longitudinal apparent leukonychia with concurrent erythronychia, reflecting vascular alterations. Histopathology reveals epidermal disorganization, dyskeratosis, and atypical keratinocytes.^2^, ^5^, ^6^, ^9^

Finally, chronic trauma, especially involving the hallux, may result in a solitary longitudinal white band. Histopathology shows hyperkeratosis, irregular acanthosis, spongiosis, and parakeratosis.^5^

## Multiple-digit leukonychia

When multiple nails are affected, inflammatory or hereditary disorders should be considered. Darier disease presents with greasy papules and plaques, sometimes associated with ocular or neuropsychiatric involvement.^10^ Nail changes include leukonychia and erythronychia (“candy cane nails”), V-shaped notching, and subungual hyperkeratosis.^5^, ^10^ Similar findings occur in Hailey-Hailey disease and in tuberous sclerosis complex.^5^ Infectious causes, such as onychomycosis, may produce pseudo-leukonychia characterized by irregular white or yellowish streaks.^5^

This case broadens the clinical spectrum of onycholemmal cysts, highlighting the need to consider them in the differential diagnosis of monodactylous longitudinal leukonychia.

## Financial support

None declared.

## Research data availability

Does not apply.

## Authors’ contributions

Maria Paula Muñoz Mc Causland: Approval of the final version of the manuscript; critical literature review; data collection, analysis and interpretation; effective participation in research orientation; intellectual participation in propaedeutic and/or therapeutic management of studied cases; manuscript critical review; preparation and writing of the manuscript; statistical analysis; study conception and planning.

Isabela Dorado Caycedo: Approval of the final version of the manuscript; critical literature review; data collection, analysis and interpretation; effective participation in research orientation; intellectual participation in propaedeutic and/or therapeutic management of studied cases; manuscript critical review; preparation and writing of the manuscript; statistical analysis; study conception and planning.

Annie Mariana Melendez: Critical literature review.

Juan Carlos López: Data collection, analysis and interpretation; intellectual participation in propaedeutic and/or therapeutic management of studied cases.

## ORCID ID

María Paula Muñoz Mc Causland: 0000-0001-7923-6670

Annie Mariana Meléndez: 0009-0003-5743-5224

Juan Carlos López Hiromi: 0000-0002-2974-2193

Isabela Dorado Caycedo: 0009-0006-0311-1269

## Conflicts of interest

None declared.
